# Visual working memory training enhances simulated mine monitoring performance: the modulating role of memory growth potential

**DOI:** 10.1186/s41235-026-00746-6

**Published:** 2026-07-17

**Authors:** Rongjuan Zhu, Xiaoliang Ma, Peiyu Liu, Qi Hui

**Affiliations:** 1 College of Management, Xi’an University of Science and Technology, Xi’an, 710054 China; 2 School of Computer Science and Technology, Anhui University, Hefei, 230601 China; 3 GEOVIS Earth Technology Co., Ltd., Hefei, 230088 China; 4 Key Laboratory for Behavior and Cognitive Neuroscience of Shaanxi Province, School of Psychology, Shaanxi Normal University, Xi’an, 710062 China

**Keywords:** Visual working memory training, Individual differences, Working memory potential, Mine safety monitoring

## Abstract

The transition to Industry 4.0 in mining has shifted operator roles from manual control to cognitive supervision, demanding heightened visual working memory (VWM) for monitoring complex data interfaces. This study examines whether adaptive VWM training improves performance in safety–critical monitoring tasks and whether individual differences in memory growth potential modulate this efficacy. Using a randomized controlled design with 59 participants, we employed a continuous metric of memory growth potential ΔK (K_6_–K_4_) to represent latent cognitive plasticity. Results show that VWM training significantly enhanced VWM capacity, with the magnitude of gains being positively predicted by an individual’s initial memory growth potential. These training benefits selectively transferred to a simulated mine monitoring task; specifically, higher growth potential positively predicted greater behavioral improvements in both gas and equipment monitoring. By contrast, no transfer occurred on a near-transfer VWM precision task. These findings suggest that VWM training yields selective, task-specific improvements in performance on simulated mining monitoring tasks, while highlighting the necessity of personalized assessment and training protocols that account for individual differences in cognitive plasticity.

## Introduction

The integration of Industry 4.0 technologies has driven a substantial shift in mining operations, transitioning from traditional manual operation to computer-based supervisory control underpinned by pervasive digitalization and intelligent automation (Wang et al., 2022). While such transformation has enhanced operational efficiency, it has also imposed heightened cognitive demands on operators in mine control rooms. In modern intelligent mine control settings, operators must monitor multiple screens and process dense, dynamic information to maintain safe and efficient system operation (Chirgwin, 2021; Wang & Huang, 2017). Some studies have indicated that this modern operational pattern substantially increases operators’ mental workload (Chirgwin, 2021; Mohammadian et al., 2022). When workload becomes excessive and exceeds available cognitive capacity, such information overload can lead to delayed decision-making or even critical human errors in dynamic visual monitoring tasks (Sakinala et al., 2023; Wickens et al., 2022). A central component of this limited cognitive capacity is visual working memory (VWM), which is the system responsible for temporarily maintaining and manipulating visual information and is essential for maintaining stable and accurate performance in demanding monitoring environments (Baddeley & Hitch, 1974; Cowan, 2001). Given that the capacity of VWM is strictly limited (Liesefeld & Müller, 2019; Luck & Vogel, 1997), investigating the potential of targeted training to improve this ability is meaningful for enhancing operator performance in complex monitoring tasks.

Substantial efforts have been invested in developing cognitive training paradigms to enhance VWM performance (Bi et al., 2020; Cai et al., 2022; Moriya, 2019; Wang & Qian, 2021). VWM training has been shown to not only improve performance in trained tasks but also produce transfer effects to untrained domains, ranging from closely related VWM measures to broader academic performance (Moriya, 2019; Zhang, 2017). However, the scope of transfer following cognitive training remains debated, with particularly mixed evidence for broad far transfer to dissimilar outcomes or general abilities (Cai et al., 2022). In cognitive training research, near transfer refers to improvements on untrained tasks that share substantial cognitive processes or task demands with the trained task, whereas far transfer refers to improvements on more dissimilar outcomes or broader abilities. Previous studies have indicated that transfer effects are more likely to occur when training tasks and transfer tasks share overlapping cognitive components (Holmes et al., 2009; Waris et al., 2015). According to the similarity-based theories, the operational demands of intelligent mine monitoring require the continuous encoding and maintenance of visual information alongside the efficient scanning of dynamic interfaces. The change detection task is particularly well suited to these demands, as it directly targets the encoding and maintenance of discrete visual representations. Crucially, Truong et al., (2022) demonstrated that adaptive change detection training can enhance both internal memory fidelity and broader perceptual skills including conjunction visual search. This finding provides a strong empirical rationale for the current study, as it implies that such training can simultaneously strengthen the storage capacity and scanning efficiency necessary for anomaly detection in complex monitoring environments. Despite this theoretical relevance, direct empirical evidence testing whether change detection-based VWM training generates applied near transfer to complex, ecologically relevant scenario remains scarce.

Moreover, even when sufficient cognitive overlap exists to support transfer, individuals may differ substantially in the extent to which they benefit from training. Recent evidence suggests that individual differences in cognitive plasticity, such as baseline working memory (WM) capacity and learning potential, play a critical role in shaping both the magnitude of training gains and the likelihood of transfer (Foster et al., 2017; Wiemers et al., 2019). Notably, research on individual differences in training effects has yielded conflicting perspectives on which populations benefit most from WM training. The compensatory account argues that individuals with lower baseline cognitive abilities stand to gain more from such training, as it helps ameliorate initial deficits (Karbach et al., 2017). Conversely, the magnification account posits that those with higher baseline capacities exhibit greater training-induced improvements, as their existing cognitive resources enable more effective utilization of training interventions (Foster et al., 2017; Lövdén et al., 2012). However, these static baseline measures often fail to capture the dynamic nature of an individual's cognitive reserve. Recent work has introduced alternative approaches that consider dynamic indicators of memory growth potential prior to training. This perspective aligns with the "testing-the-limits" paradigm, which suggests that assessing cognitive performance under near-maximal task demands provides a more sensitive measure of an individual's latent cognitive plasticity (Kliegl et al., 1989). In this framework, memory growth potential reflects an individual’s ability to mobilize additional cognitive resources when task demands approach or exceed their initial capacity limits. This conceptualization is grounded in the cognitive architecture of VWM, where capacity is typically limited to 3 to 5 items (Luck & Vogel, 1997). Accordingly, 4-item loads approach the capacity boundary for most individuals, while 6-item loads typically exceed it. For individuals with low growth potential, presenting items beyond their capacity may induce interference in memory maintenance, which leads to stable or even reduced performance. Conversely, those whose capacity limit is not reached at load 4 can continue to allocate additional resources as the load increases to six items, thus exhibiting higher latent cognitive plasticity (Wang & Ku, 2018). For example, Wang & Ku, (2018) utilized VWM capacity increments across increasing load conditions (from K_4_ to K_6_) as an index of memory growth potential. They demonstrated that anodal stimulation of the right dorsolateral prefrontal cortex selectively enhanced performance in individuals with higher growth potential. While this provides evidence for the role of latent cognitive plasticity in neuromodulation, it remains unexplored whether this dynamic metric can serve as a continuous predictor of behavioral VWM training outcomes and their applied near transfer to simulated intelligent mining scenarios.

To address these concerns, the current study aimed to explore the efficacy of adaptive VWM training in improving VWM capacity and investigate the extent to which such training gains generalize to untrained outcomes, including VWM precision and performance in a simulated mine monitoring task. Consistent with established paradigms (Luck & Vogel, 1997; Xu et al., 2018), a color-based change detection task was employed for training, while an adaptive visual search task served as the active control (Harrison et al., 2013; Moriya, 2019). Crucially, we employed the "testing-the-limits" framework (Kliegl et al., 1989) to operationalize memory growth potential as a continuous index calculated from the capacity increment from load 4 to load 6 (ΔK = K_6_–K_4_). This dynamic metric serves as a behavioral proxy for latent cognitive plasticity, reflecting an individual's ability to mobilize additional resources when task demands approach their capacity limits (Wang & Ku, 2018). By integrating this perspective, we aimed to examine how individual differences in latent plasticity influence both training-related improvements and the applied near transfer of gains to ecologically valid scenarios.

Based on this theoretical framework, we formulated the following a priori hypotheses:

1. The adaptive VWM training group will exhibit significantly greater improvements in both VWM capacity and simulated mine monitoring performance compared to the active control group. By contrast, we hypothesized no significant training-related transfer to the VWM precision task, given its mismatched response characteristics and distinct cognitive demands relative to the training task.
2. VWM training gains would be significantly predicted by individual memory growth potential, such that individuals with higher potential scores tend to exhibit larger training improvements.
3. Memory growth potential will positively predict the magnitude of applied near transfer to simulated mine monitoring performance, such that individuals with higher cognitive plasticity demonstrate larger behavioral benefits.

## Experiment

### Methods

#### Participants

We recruited 60 mining engineering students (Mean age = 20.87 years, *SD* = 2.67; 45 men, 15 women) for the experiment. All participants met the following inclusion criteria: (a) right-handedness, (b) normal or corrected-to-normal vision, and (c) no prior knowledge of the experimental hypotheses. Due to technical equipment failure, pretest data from one participant were excluded. Consequently, the final analysis included 59 participants. All participants provided written informed consent and were compensated for their time and effort upon completion of the study.

#### Pre- and posttest

##### Visual working memory capacity

**Fig. 1:**
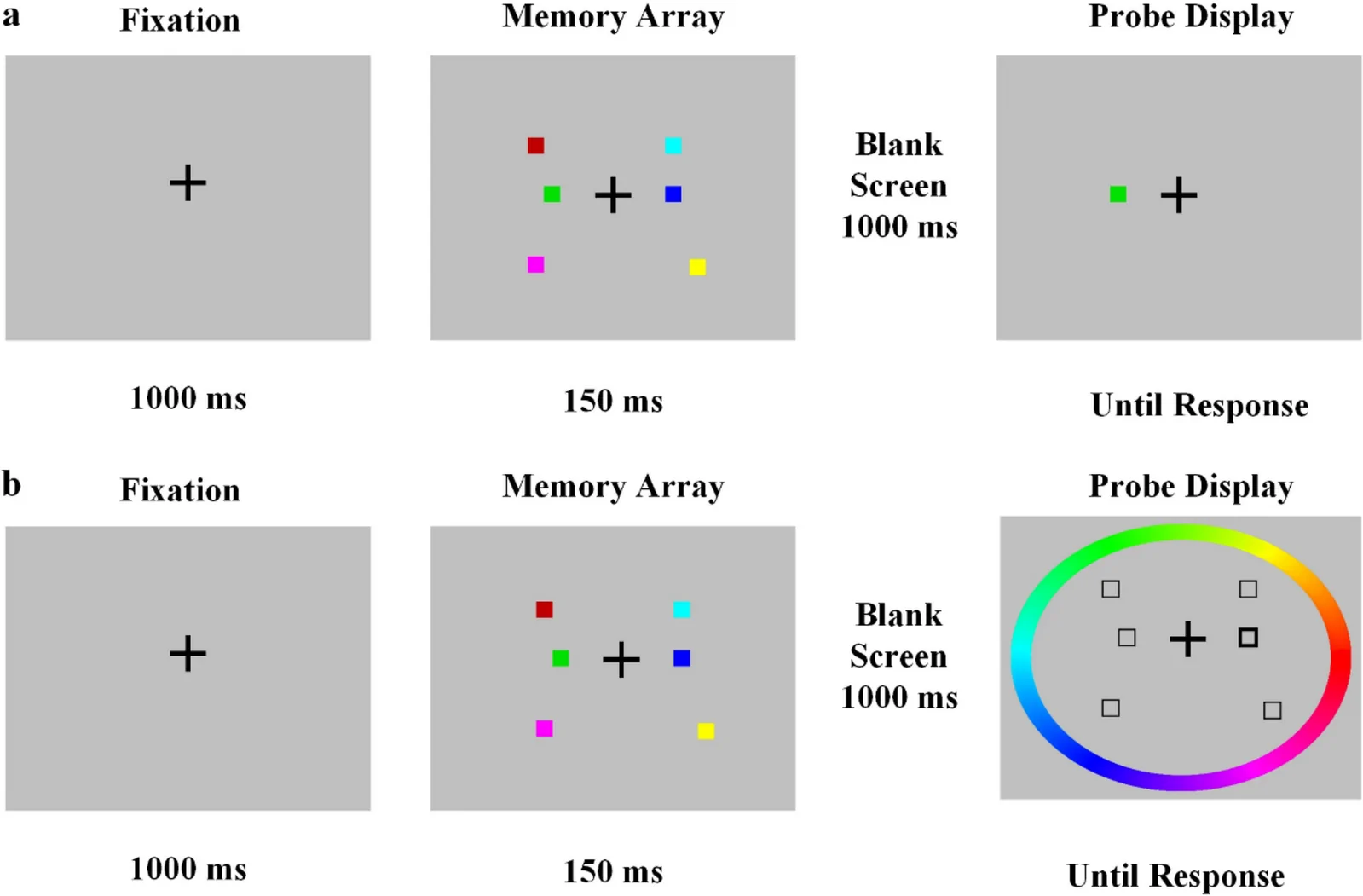
A schematic of visual working memory capacity (**a**) and precision (**b**) tasks measure

Participants performed a change detection task to measure VWM capacity. Each trial began with a central fixation point that remained visible throughout. After a 1000-ms delay, a memory array consisting of 4 or 6 colored squares (0.68° × 0.68° visual angle, minimum center-to-center spacing = 2°) was presented for 150 ms against a gray background (RGB: 192,192,192). The squares appeared in seven highly discriminable colors (red [255,0,0], purple [255,0,255], yellow [255,255,0], cyan [0,255,255], blue [0,0,255], green [0,255,0], and orange [255,165,0]). Following a 1000-ms retention interval, a single probe square appeared at one of the previously occupied locations, and participants judged whether its color matched the original item at that position (Fig. 1a). Trial types (set sizes, probe match/mismatch) were presented in pseudorandom order.

##### Visual working memory precision

Participants performed a color recall task with stimulus parameters identical to the capacity task (including array size, presentation duration, and color set), except for the response modality. Following the 1000-ms retention interval, instead of a binary change detection judgment, participants reproduced the exact color of a cued item from the memory array using a continuous color wheel (Fig. 1b). The wheel displayed 360° hue variations of the target color space, requiring precise selection of the memorized color's hue, saturation, and brightness. Response error was calculated as the angular deviation (in degrees) between the selected and original colors, with lower values indicating higher precision.

##### Simulated mine monitoring tasks

**Fig. 2:**
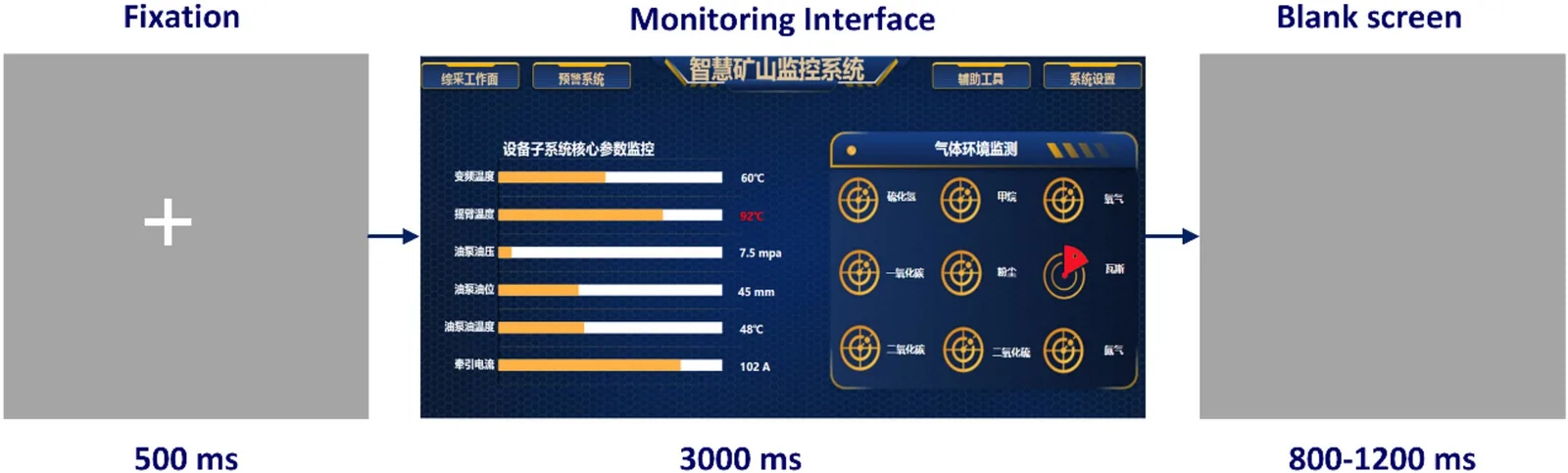
Experimental procedure of simulated mine monitoring task

A simplified version of the type of intelligent mine monitoring systems used for coal mines was adopted for this experiment, comprising fully mechanized equipment operation status monitoring in an intelligent coal mining face and underground gas environment monitoring tasks (Fig. 2). Participants were required to detect abnormalities in equipment parameters and gas concentrations. The left panel, designated as the equipment subsystem, displays six critical parameters (frequency conversion temperature, rocker arm temperature, oil pump pressure, oil pump level, oil pump temperature, and traction current) as dynamic bar graphs accompanied by their corresponding numerical values. During an equipment malfunction, the affected bar graph elongates and flashes, with its numerical value displayed in a flashing red color to indicate the abnormality. Participants were instructed to respond immediately by pressing the “F” key on a standard QWERTY keyboard. The right panel, designated as the gas monitoring section, presents nine gas indicators (hydrogen sulfide, carbon monoxide, carbon dioxide, methane, dust, oxygen, sulfur dioxide, nitrogen, and gas) as gold circular icons. When any gas concentration became hazardous, its icon transformed into a flashing red sector, participants were instructed to press the “J” key to respond to these gas-related anomalies.

All experimental stimuli were presented using PsychoPy 2023.2.3 software on a 1920 × 1080 resolution monitor, with participants seated approximately 60 cm from the screen. Each formal trial commenced with a white fixation cross (500 ms) presented at the center of a gray screen to stabilize participants’ attention. Following the fixation cross, the mine monitoring interface appeared, displaying dynamic updates of equipment parameters (e.g., subtle fluctuations in bar graph lengths) and gas indicators for a total duration of 3000 ms. The interface remained visible until a participant responded, or disappeared automatically after a maximum of 3000 ms if there was no response. Each trial was followed by a blank screen interval of 800–1200 ms (randomly jittered) to minimize carryover effects between trials. There are 80 trials in the experiment, including 60% normal trials, 30% abnormal trials and 10% false alarm trials. False alarm trials were included to simulate the “ambiguous signals” commonly encountered in real-world mining monitoring. In the false alarm trials, either the bar graphs and numerical values in the left equipment panel flashed (without concurrent color changes or abnormal length variations) or the circular icons in the right gas monitoring panel flashed (without transforming into red sectors). No response was required from participants during normal trials or false alarm trials. Prior to the formal experiment, participants completed 15 practice trials to familiarize themselves with the task requirements and response protocols.

#### Training program

The VWM capacity training protocol employed an adaptive change detection task identical to the pre- and posttest except for the following modifications (Fig. 1a). The number of colored squares was dynamically adjusted based on participants' task performance during training: Participants complete 6 blocks per training session, with each block containing 30 trials, and the initial difficulty of the first block is set to 4 colored squares; after each block, the difficulty of the subsequent block is determined according to the participant's accuracy, such that if the accuracy in the current block reaches 90% or higher, the number of to-be-memorized colored squares in the next block is increased to enhance training difficulty, if the accuracy falls between 75% and 90%, the difficulty of the next block remains unchanged, and if the accuracy is lower than 75%, the difficulty level decreased in the next block. The minimum and maximum difficulties set to 1 and 12 colored squares, respectively.

The entire training intervention consisted of six sessions conducted over six consecutive workdays. Each session (180 trials) lasted approximately 20 to 25 min, resulting in a total active training duration of 120 to 150 min per participant. This intensive short-term schedule was adapted from established protocols in VWM literature (Moriya, 2019; Truong et al., 2022), which have demonstrated that six to seven adaptive sessions are sufficient to elicit significant cognitive plasticity and transfer effects. To ensure high adherence, participants received daily reminders and were required to complete all sessions in a quiet laboratory environment. As a result, all 59 participants who started the intervention successfully completed the entire 6-day training program, representing a 100% adherence rate with no dropouts during the training process.

We adopted a visual search task training protocol in the active control group. In this adaptive task, participants were required to detect an orientation target among distractors. Task difficulty, defined by the number of items in the search array, was dynamically adjusted based on performance. Participants completed 6 blocks per session, with 30 trials per block. The initial difficulty was set to a 2 × 2 array (4 items). After each block, the difficulty of the next block was determined by the participant's accuracy and reaction time (RT): If accuracy was ≥ 80% and RTs were < 1000 ms, the array size increased; if accuracy was ≥ 80% but RTs were between 1000 and 1500 ms, the difficulty remained unchanged; and if accuracy was < 80% or RTs exceeded 1500 ms, the difficulty decreased. The minimum and maximum difficulties were set to 4 (2 × 2 array) and 64 (8 × 8 array) items, respectively.

### Data analysis

All statistical analyses were conducted using linear mixed-effects models (LMMs). The mixed-effects models included fixed effects of experimental conditions and random intercepts for participants to account for individual variability. Continuous memory growth potential scores were grand mean-centered to reduce multicollinearity and facilitate the interpretation of interaction effects. To align the power assessment with the final analytic strategy, we conducted simulation-based power analyses based on the final models. The simulations used the actual study design, including 59 participants, two groups (training: n = 30; control: n = 29), and two test times (pretest and posttest). For the VWM training effect, power was estimated for the group × time interaction in the model K value ~ group × time + (1 | participant). Using 10,000 Monte Carlo simulations with α =.05, the estimated power to detect the observed group × time interaction was greater than.99. For the moderation analysis, K value gain was predicted by group, mean-centered memory growth potential, and their interaction. The estimated power to detect the observed group × memory growth potential interaction was.816.

To analyze the effects of VWM training, the LMMs was conducted. The model included group (training vs. control), test time (pretest vs. posttest) as fixed effects, and participant identity as a random intercept. K values were calculated using Cowan’s, (2001) formula: K = S × (H−FA) (S is the size of the memory array, HR is the hit rate, and FA is the false alarm rate). To test whether memory growth potential moderated training-related gains, a hierarchical linear regression was performed with training gain (posttest K − pretest K) as the dependent variable. Predictors included group (training vs. control), mean-centered memory growth potential, and their two-way interaction.

For the transfer effect on VWM precision performance, the LMM included load type (4 vs. 6 colored squares, within-subjects), test time (pretest vs. posttest, within-subjects), and group (training vs. control, between-subjects) as fixed effects. To explore individual differences in transfer effects, mean-centered continuous memory growth potential scores and its higher-order interactions with group and time were additionally integrated into the model. Precision was quantified as the standard deviation (SD) of the response error, calculated as the circular difference (in degrees) between the participant's reported color and the true color of the target stimulus across trials. A lower SD indicates higher precision. To clarify the moderating role of memory growth potential on precision transfer, we focused on the three-way interaction of group × time × memory growth potential within the same LMM framework.

For the transfer effect on mine monitoring performance, two key metrics were analyzed: the discriminability index (*d'*) and RT. The formula was *d'* = Z _(Hit Rate)_ – Z _(False Alarm Rate)_, where the hit rate was the proportion of correct responses to abnormal trials, and the false alarm rate was the proportion of incorrect responses to normal trials. RT was defined as the mean reaction time taken to respond correctly to abnormal trials. For both the equipment monitoring task and the gas monitoring task, LMMs models were used to analyze transfer effects, with test time (pretest vs. posttest) and group (training vs. control) as fixed effects. To test whether memory growth potential moderated transfer effects on *d′* and RT, mean-centered potential score and its interactions with group and time were added to the models as fixed-effect predictors. Finally, to quantify participants' response bias and impulsive responding tendencies toward ambiguous signals, we calculated a lure error rate for each participant. Here, "lure trials" specifically refer to the false alarm trials described in the task section (i.e., the 10% of trials with flashing but non-hazardous signals that required no response). The lure error rate was defined as the proportion of incorrect responses made to these trials. Separate LMMs were conducted for the equipment monitoring task and the gas monitoring task. Each model included test time (pretest vs. posttest) and group (training vs. control) as fixed effects. To examine whether memory growth potential modulated changes in lure error rates, the models were extended to include mean-centered potential score and its interactions with group and time.

### Results

#### Training effect

**Fig. 3:**
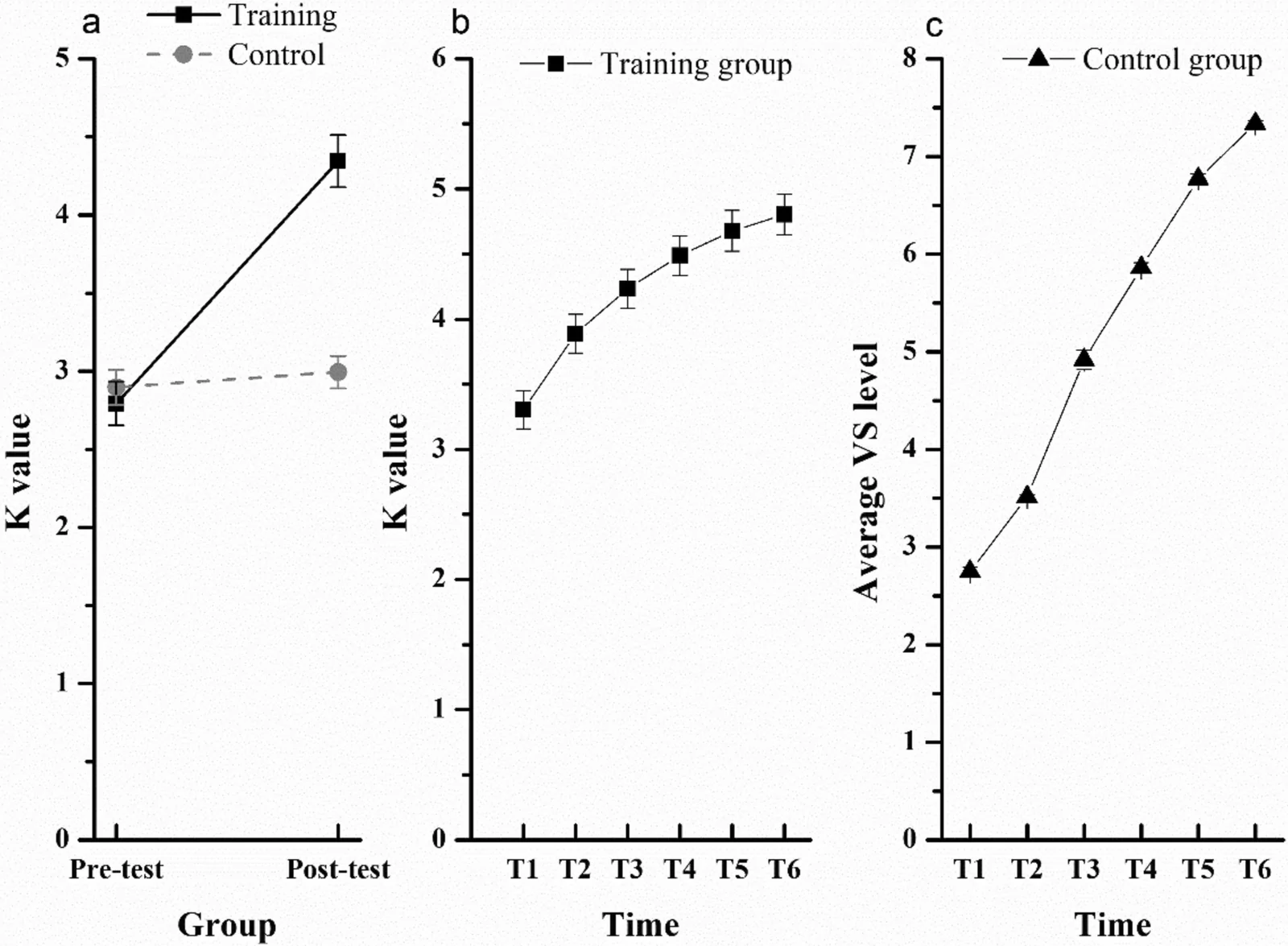
Effects of visual working memory capacity training on performance. **a** Pretest and posttest *K* values of training group and control group; **b** temporal changes in *K* value of training group. **c** Temporal changes in visual search difficulty level of control group

The LMM revealed a significant group × time interaction effect (*β* = 1.456, SE = 0.124, *t* = 11.768, *p* <.001). Simple effect analyses indicated that the control group showed no significant pretest-to-posttest change in VWM capacity (*ΔK* = 0.097, SE = 0.088, *t* = 1.098, *p* =.272). In contrast, the training group exhibited a substantial and highly significant increase in VWM capacity from pretest to posttest (*ΔK* = 1.553, SE = 0.152, *t* = 10.219,* p* <.001) (Fig. 3a).

Furthermore, we analyzed the temporal dynamics during the training phase. The training group exhibited a clear upward trend in VWM capacity (*K* value) across days (Fig. 3b). Correspondingly, the increasing difficulty level of the visual search task for the active control group (Fig. 3c) verified their sustained engagement throughout the training period.

**Fig. 4:**
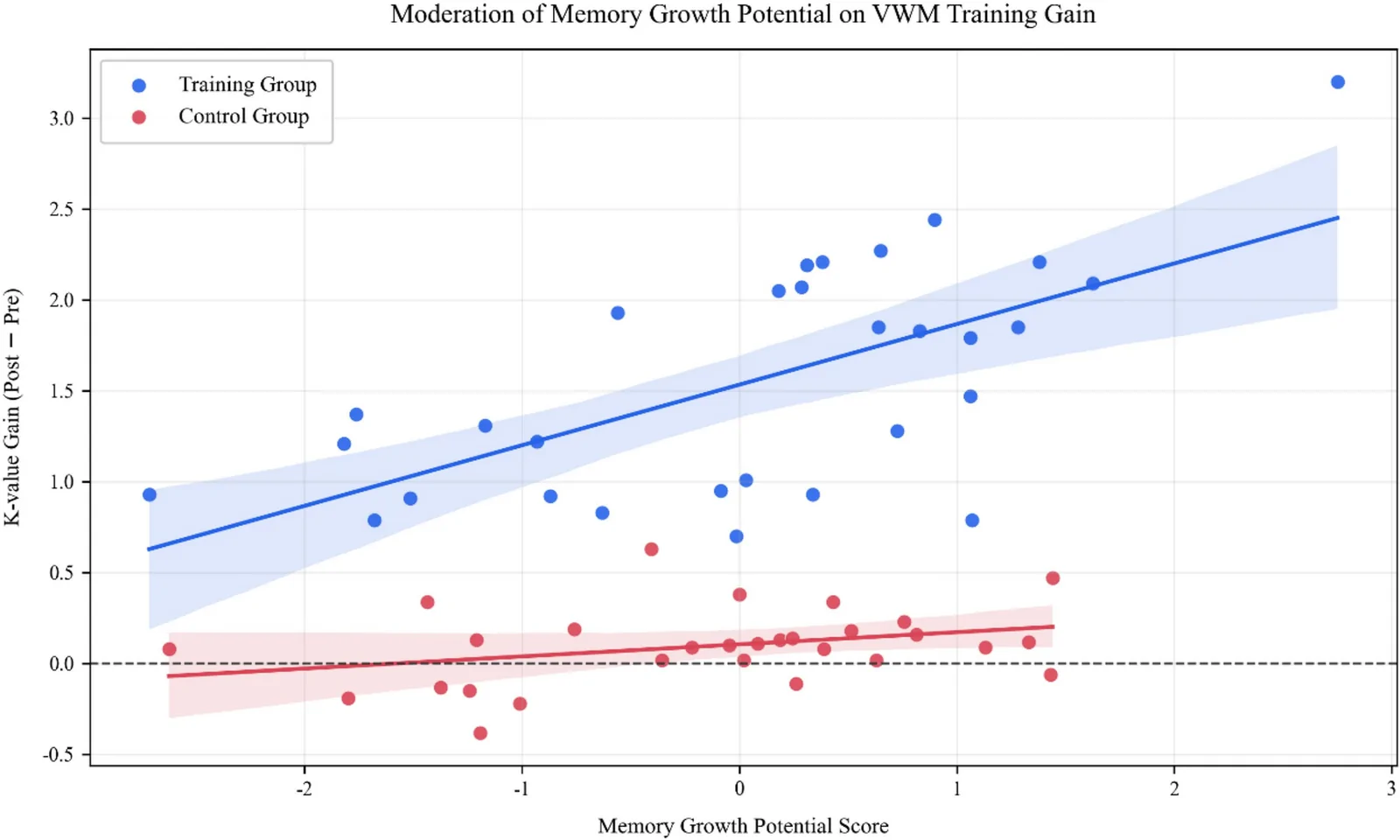
Memory growth potential differentially predicts VWM gains across training and control groups

To examine whether memory growth potential moderated training gains, a hierarchical linear regression was conducted with training gain (posttest K − pretest K) as the dependent variable. The model included group (training vs. control), mean-centered memory growth potential, and their two-way interaction as predictors. The overall regression model was significant (*R*^*2*^ = 0.819, *F* (3, 55) = 83.22, *p* <.001). Critically, the interaction between group and memory growth potential was significant (*β* = 0.267, SE = 0.091, *t* = 2.927, *p* =.005), indicating that the association between memory growth potential and training gain differed significantly between groups. Follow-up within-group simple regression analyses revealed that memory growth potential was positively associated with training gain in the training group (*r* = 0.641, *β* = 0.333, *p* <.001), whereas no significant association was observed in the control group (*r* = 0.321, *β* = 0.067, *p* =.090) (Fig. 4).

#### Transfer effect

##### VWM precision performance

The LMM revealed a significant main effect of load type (*β* = 2.278, SE = 0.533, *t* = 4.275, *p* <.001). Post hoc interpretation confirmed that VWM precision was significantly higher under the low-load condition (*M* = 22.07) than under the high-load condition (*M* = 26.29). No significant main effects of test time (*β* = − 1.047, SE = 3.843, *t* = − 0.272, *p* =.785) or group (*β* = 3.776, SE = 3.981, *t* = 0.948, *p* =.343) were observed. Critically, the group × time interaction was nonsignificant (*β* = 0.588, SE = 5.385, *t* = 0.109, *p* =.913). The three-way interaction of load type × group × time also failed to reach significance (*β* = − 0.124, SE = 1.056, *t* = − 0.117, *p* =.907) (Table 1). These results suggest that the VWM capacity training program did not lead to significant improvements in VWM precision.

**Table 1:** The training effect and transfer effect

Task	Training group			Control group		
	Pre	Post	Gain	Pre	Post	Gain
VWM capacity	2.79(0.77)	4.34(0.91)	1.55(0.63)	2.89(0.59)	2.99(0.56)	0.10(0.21)
Precision (load 4)	22.82(6.50)	23.13(5.64)	0.31(2.98)	20.97(3.23)	21.37(3.71)	0.40(2.09)
Precision (load 6)	26.47(7.57)	26.72(7.83)	0.25(1.45)	25.94(5.89)	26.02(6.08)	0.08(1.30
Equipment (*d′*)	1.67(0.33)	1.91(0.44)	0.24(0.35)	1.66(0.29)	1.76(0.26)	0.09(0.22)
Equipment (RT)	865(69)	834(62)	−31(63)	869(45)	861(39)	−8(22)
Gas (*d′*)	2.28(0.59)	2.53(0.58)	0.25(0.51)	2.35(0.77)	2.33(0.68)	−0.02(0.35)
Gas (RT)	798(87)	755(97)	−43(22)	789(83)	790(86)	1(9)

Values in parentheses are standard deviations (SD)

To further investigate whether memory growth potential moderated precision transfer, we focused on the group × time × memory growth potential interaction. This three-way interaction was nonsignificant (*β* = 0.394, SE = 4.928, *t* = 0.080, *p* =.936). No other interactions involving memory growth potential reached statistical significance (all *ps* >.05). This indicates that the training did not differentially transfer to precision performance gains as a function of individual growth potential.

#### Simulated mine monitoring tasks performance

##### Discriminability index (*d'*) and RT of the equipment monitoring task

**Fig. 5:**
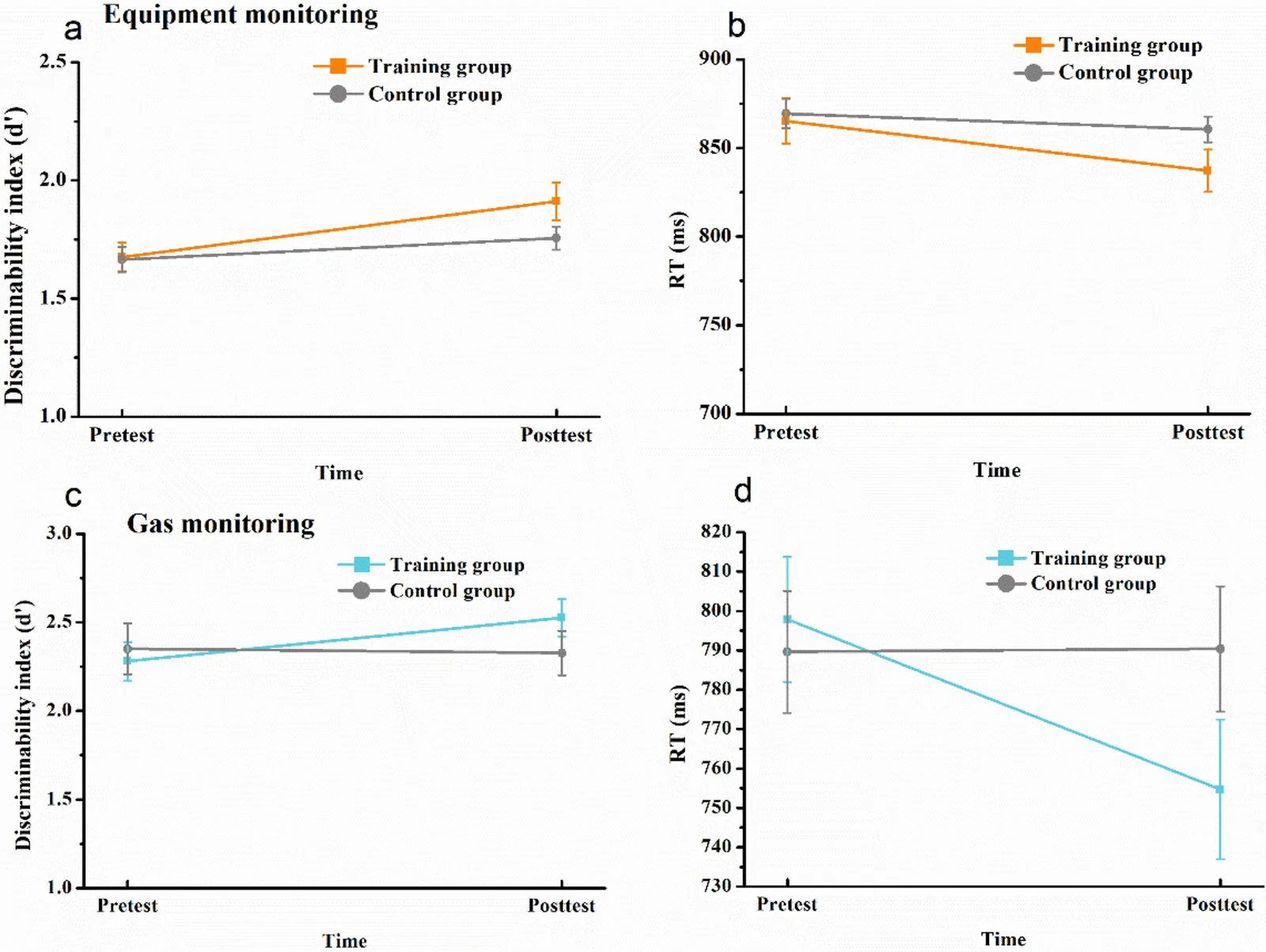
Transfer Effects of VWM training on monitoring task performance. **a** Pretest and posttest performance of discriminability index in equipment monitoring task for training and control group; **b** pretest and posttest performance of reaction time (RT) in equipment monitoring task for training and control group; **c** pretest and posttest performance of discriminability index in gas monitoring task for training and control group; **d** pretest and posttest performance of RT in gas monitoring task for training and control group

For the discriminability index (*d′*), the LMM showed no significant main effect of time (*β* = 0.092, SE = 0.055, *t* = 1.668, *p* =.095) and group (*β* = 0.011, SE = 0.088, *t* = 0.123, *p* =.902). Additionally, the time × group interaction was nonsignificant (*β* = 0.144, SE = 0.077, *t* = 1.867, *p* =.062) (Fig. 5a).

For the RT, the LMM revealed no significant main effect of time (*β* = − 8.828, SE = 8.858, *t* = − 0.997, *p* =.319), even though posttest RT was numerically faster than pretest RT. The main effect of group was nonsignificant (*β* = − 4.248, SE = 14.412, *t* = − 0.2948, *p* =.768), and the time × group interaction was also nonsignificant (*β* = − 22.172, SE = 12.422, *t* = − 1.785, *p* =.074) (Fig. 5b).

##### The effect of individual's memory growth potential

To explore how individual memory growth potential influences training transfer, LMMs were performed on *d′* gain (posttest *d′* − pretest *d′*) and RT gain (posttest RT−pretest RT), with group (control vs. training), centered memory growth potential, and their interaction as fixed effects, and participant identity as a random effect.

For *d′* gain, the LMM yielded a significant group × potential interaction (*β* = 0.137, SE = 0.068, *t* = 1.995, *p* =.046). Simple effect analyses demonstrated that in the training group, memory growth potential was significantly positively associated with *d′* gain (*β* = 0.093, SE = 0.046, *t* = 2.014, *p* =.044, *R*^*2*^ = 0.536), indicating greater *d′* improvements for individuals with higher potential. In contrast, there was no significant association between potential and *d′* gain in the control group (*β* = − 0.044, SE = 0.041, *t* = − 1.087, *p* =.277, *R*^*2*^ = 0.503).

For RT gain, a significant group × potential interaction was also observed (*β* = − 22.007, SE = 10.836, *t* = − 2.031, *p* =.042). Simple effects analysis showed that in the training group, higher potential was significantly associated with greater RT reduction (*β* = − 19.735, SE = 7.269, *t* = − 2.715, *p* =.007, *R*^*2*^ = 0.557). Conversely, in the control group, higher potential was significantly linked to less RT reduction (*β* = 2.272, SE = 0.659, *t* = 3.448, *p* <.001, *R*^*2*^ = 0.487).

##### Discriminability index (*d'*) and RT of the gas monitoring task

For *d′*, the LMM revealed no significant main effects of time (*β* = − 0.021, SE = 0.082, *t* = − 0.256, *p* =.798) or group (*β* = − 0.068, SE = 0.171, *t* = − 0.399, *p* =.689). Critically, a significant time × group interaction was observed (*β* = 0.266, SE = 0.115, *t* = 2.311, *p* =.021). Simple effect analyses showed that the training group exhibited a significant increase in *d′* from pretest (*M* = 2.280, *SD* = 0.594) to posttest (*M* = 2.525, *SD* = 0.576) (*p* =.002), whereas no such improvement was observed in the control group (*p* =.798) (Fig. 5c).

For RT, the LMM also yielded no significant main effects of time (*β* = 0.724, SE = 3.138, *t* = 0.231, *p* =.817) or group (*β* = 8.142, SE = 23.060, *t* = 0.353, *p* =.724). However, a significant time × group interaction was found (*β* = − 43.891, SE = 4.400, *t* = −9.974, *p* <.001). Simple effect analyses showed that the training group exhibited a significant reduction in RT from pretest (*M* = 797 ms, *SD* = 86) to posttest (*M* = 754 ms, *SD* = 97) (*p* <.001), while no significant difference between pretest and posttest was observed in the control group (*p* =.817) (Fig. 5d).

##### The effect of individual's memory growth potential

For *d′* gain, the LMM showed a significant main effect of group (*β* = 0.237, SE = 0.103, *t* = 2.300, *p* =.021), indicating larger *d′* improvements in the training group (*M* = 0.245, *SD* = 0.515) than in the control group. The main effect of potential was nonsignificant (*β* = 0.057, SE = 0.073, *t* = 0.780, *p* =.435). A marginally significant group × potential interaction was observed (*β* = 0.185, SE = 0.094, *t* = 1.958, *p* =.050), reflecting a trend that higher potential was associated with larger *d′* gains in the training group but not in the control group.

For RT gain, the LMM revealed a highly significant main effect of group (*β* = − 42.585, SE = 3.574, *t* = − 11.915, *p* <.001), confirming greater RT reduction in the training group relative to the control group. The main effect of potential was nonsignificant (*β* = − 1.718, SE = 2.519, *t* = − 0.682, *p* =.495). Importantly, a significant group × potential interaction was found (*β* = −10.014, SE = 3.270, *t* = − 3.062, *p* =.002; Fig. 5 d). Simple effects analyses indicated that in the training group, higher memory growth potential was significantly associated with greater RT reduction (*β* = − 11.732, SE = 1.991, *t* = − 5.893, *p* <.001, *R*^*2*^ = 0.704), explaining 70.4% of the variance in RT gain. In the control group, the association between potential and RT reduction was nonsignificant (*β* = −1.718, SE = 0.928, *t* = −1.851, *p* =.495, *R*^*2*^ = 0.500).

##### False alarm trials (lure trials) performance

For the equipment monitoring task, the LMM revealed a nonsignificant time × group interaction, *β* = −0.049, SE = 0.053, *t* = −0.933, *p* =.351. A subsequent LMM showed that there was nonsignificant group × potential interaction, *β* = 0.036, SE = 0.045, *t* = 0.802, *p* =.422. Descriptive data for all groups are presented in Table 2.

**Table 2:** Descriptive statistics for lure error rate in the equipment and gas monitoring tasks

Tasks	group	Pretest lure error rate	Posttest lure error rate	Gain
Equipment	Training	0.26(0.20)	0.19(0.18)	−0.07(0.19)
	Control	0.27(0.23)	0.25(0.21)	−0.02(0.21)
Gas	Training	0.21(0.19)	0.12(0.13)	−0.09(0.17)
	Control	0.22(0.17)	0.21(0.16)	−0.01(0.18)

Values in parentheses represent standard deviations

For the gas monitoring task, the LMM revealed a nonsignificant time × group interaction, *β* = −0.083, SE = 0.046, *t* = −1.822, *p* =.068. A subsequent LMM showed that there was nonsignificant group × potential interaction, *β* = 0.033, SE = 0.035, *t* = 0.943, *p* =.346. Descriptive data for all groups are presented in Table 2.

## Discussion

The present study aimed to address the cognitive demands of Industry 4.0-driven mining digitalization by examining whether adaptive VWM training enhances VWM capacity and simulated mine monitoring performance, and whether these effects are moderated by an individual’s memory growth potential. Our results suggest that adaptive VWM training can lead to improved performance on the same task, though these benefits did not appear to transfer to VWM precision. This pattern aligns with the theoretical perspective that transfer effects are most likely to occur when training and transfer tasks share overlapping cognitive components (Holmes et al., 2009; Waris et al., 2015). In this study, the training task was structurally identical to the VWM capacity assessment, featuring the same stimuli and encoding phase. But it was somewhat dissimilar to the precision task in terms of response requirements. Therefore, the observed improvements may reflect highly specific near-transfer effects facilitated by this cognitive overlap, rather than a generalized enhancement of the broader VWM system.

From a neurocognitive perspective, this observed divergence between capacity and precision is plausible given that these two may represent dissociable components of VWM supported by distinct neural architectures. Previous neuroimaging research has indicated that capacity limitations are primarily associated with sustained activation in parietal regions responsible for attentional allocation, while recall precision depends more critically on the representational fidelity in occipital sensory areas (Wang et al., 2019, 2020; Zhao et al., 2020). Converging evidence from training studies further demonstrates that capacity-focused interventions may induce plastic changes in parietal networks that enhance the quantity of stored items without concurrently improving their sensory resolution (Cai et al., 2022). Consequently, our findings highlight the high functional specificity of VWM training, suggesting that enhancing the quantity of mental representations does not inherently improve their quality, particularly when the training environment does not explicitly challenge the fidelity of stored information.

Despite the lack of transfer to precision, training-related improvements in VWM capacity produced task-specific transfer effects in the simulated mine monitoring tasks. Although these monitoring tasks feature a 3000-ms stimulus duration, which might initially appear to reduce the demand for active maintenance, successful anomaly detection in digitized industrial interfaces requires operators to continuously update and compare fluctuating on-screen parameters against maintained mental representations of safety thresholds. The strategic choice of a change detection training paradigm is highly relevant to this cognitive demand because it targets the core mechanisms of updating and maintaining discrete items in VWM (Truong et al., 2022). This functional alignment explains the significant group-level transfer observed in the gas monitoring task, where training enhanced both discriminability (*d'*) and response speed. The shared reliance on feature binding and change detection processes between the training task and the dynamic color-coded monitoring demands of the gas task suggests that these effects reflect applied near transfer rather than broad far transfer (Holmes et al., 2009; Waris et al., 2015).

The differential transfer effects observed between the gas and equipment monitoring tasks may be further explained by varying attention-guiding potencies across visual feature types and their corresponding cognitive processing costs. The gas monitoring task requires tracking nine distinct visual indicators, which imposes a relatively high cognitive load that approaches individual VWM capacity limits. Its heavy reliance on color-coded icons likely benefits from the inherent strength of color-based attentional selection. According to Qian et al. (2020), involuntary color-based attention can modulate VWM performance irrespective of task requirements, which may allow training-related capacity gains to transfer effectively and contribute to the observed group-level benefits in the gas monitoring task. In contrast, the equipment monitoring task presents six parameters as bar graphs and may rely more on spatial orientation processing and quantitative comparison, which are cognitive components that were minimally targeted in the present color-based VWM training. As suggested by Arnicane & Souza, (2021), deploying feature-based attentional cues within complex spatial contexts is cognitively demanding and requires additional processing resources. The lower perceptual and cognitive overlap between training and equipment monitoring task demands, together with the higher resource cost of non-color feature processing, accounts for the absence of significant overall transfer at the group level. Consequently, transfer to such resource-demanding monitoring tasks appears to be constrained by a higher cognitive threshold, with performance benefits primarily observable among individuals with higher memory growth potential.

The role of individual differences in shaping transfer effects has been a focus of ongoing investigation, with prior research offering valuable insights into how factors such as baseline WM capacity, initial performance on transfer tasks, and demographic traits influence training generalization (Katz et al., 2020; Wiemers et al., 2019). Crucially, our findings suggest that transfer to simulated mine monitoring tasks may not be uniform, but rather appears to be continuously modulated by an individual’s memory growth potential. Expanding upon static baseline measures, we employed ΔK (K_6_–K_4_) as a continuous behavioral proxy for latent cognitive plasticity, which reflects the capacity to mobilize additional resources under increasing cognitive demand. In the equipment monitoring task, while no group-level improvement was observed, higher memory growth potential significantly predicted greater *d'* gains and larger RT reductions specifically within the training group. This pattern suggests that for tasks characterized by lower perceptual overlap and higher spatial and quantitative processing demands, latent plasticity tends to play an important supportive role in deploying acquired VWM training gains. Regarding the gas monitoring task, memory growth potential similarly emerged as a significant predictor of training-related benefits, which was evidenced by a robust association with RT reductions alongside a marginal tendency toward improvements in *d'*. This pattern indicates that individuals with higher latent plasticity still exhibit a relative advantage in optimizing behavioral gains. Notably, the divergent pattern observed in the control group, where higher potential was associated with smaller RT reductions, provides further evidence suggesting that the enhancements in the training group may likely stem from the synergistic effect of expanded VWM resources and inherent plasticity rather than mere practice effects.

The analysis of performance on false alarm trials provides important boundary conditions for the observed transfer effects. The absence of significant group-level and group–memory growth potential interactions across both tasks indicates that lure trial performance changes did not differ systematically between the training and control groups, nor were they modulated by individual plasticity. These findings suggest that the transfer mechanism may be characterized as a refinement of perceptual discrimination for target signals, rather than a generalized enhancement of response inhibition under uncertainty. While the training facilitated the identification of target-related features, it did not appear to bolster the cognitive control mechanisms required to suppress responses when faced with high-conflict or ambiguous stimuli, implying that the observed training gains were driven by improved sensitivity to signal-defining characteristics rather than a fundamental shift in inhibitory control.

Building upon these mechanistic insights, the observed influence of memory growth potential on transfer effects indicates that VWM training could have promising applications in safety–critical domains that demand continuous monitoring of multiple visual signals, such as mining operations or air traffic control. This aligns with established findings linking WM capacity to situational awareness in pilots (Cak et al., 2020), performance efficiency in air traffic controllers (Shorrock, 2017) and construction workers (Liko et al., 2020). The current study extends these literatures by highlighting the critical role of individual differences in cognitive training. This suggests that accounting for individual differences in cognitive plasticity during training may lead to more effective and personalized approaches to personnel selection and professional development in safety–critical fields.

Despite these insights, several limitations warrant caution. First, the simulated monitoring task might not have sufficiently taxed VWM active maintenance, as stimuli remained visible during the response window. Additionally, the present study did not establish a direct baseline correlation between standard VWM capacity and monitoring performance, which limits our ability to precisely quantify the concurrent validity of the transfer task. Future studies could adopt occlusion paradigms to more strictly isolate pure VWM processing demands and incorporate direct validity measures to further calibrate the VWM load of various industrial monitoring interfaces. Second, the training dosage and follow-up were relatively limited in both duration and scope. Future research should employ preregistered designs with a larger training dose or extended intervention periods to enhance the replicability and investigate whether such interventions yield more robust and longer-lasting transfer effects. Furthermore, as suggested by the current findings, future studies could incorporate additional training arms targeting VWM precision to complement the current focus on VWM capacity. Finally, since the present findings are based solely on a student sample, generalizability to professional operators in real high-stakes operational environments should be treated with caution. Future research could prioritize longitudinal training interventions among professional cohorts, alongside more complex and high-demand monitoring scenarios, to further clarify the boundary conditions of VWM training transfer.

## Conclusion

The current study demonstrates that the efficacy of adaptive VWM training is significantly modulated by an individual’s memory growth potential. While no transfer was observed for VWM precision, memory growth potential emerged as a consistent continuous predictor of training-related gains in simulated mine monitoring tasks, which can be interpreted as applied near transfer. Specifically, individuals with higher latent plasticity exhibited greater behavioral improvements in both gas and equipment monitoring. These findings underscore the nuanced role of individual plasticity in training and transfer effects, suggesting that the benefits of such interventions may be specific to certain cognitive profiles and task demands. This highlights the need to account for individual differences rather than assuming a universal training benefit when designing interventions for safety–critical domains.

## Data Availability

The datasets used and/or analyses are available from the corresponding author on reasonable request.

## Declarations

### Declaration of AI-assisted technologies

The authors used AI-assisted tools to support language editing and organizational refinement. All text revised with AI assistance was carefully reviewed and verified by the authors, who take full responsibility for the accuracy and integrity of the final manuscript.
